# Micro/Nanofibre Optical Sensors: Challenges and Prospects

**DOI:** 10.3390/s18030903

**Published:** 2018-03-18

**Authors:** Limin Tong

**Affiliations:** 1 State Key Lab of Modern Optical Instrumentation, College of Optical Science and Engineering, Zhejiang University, Hangzhou 310027, China; phytong@zju.edu.cn; 2 Collaborative Innovation Center of Extreme Optics, Shanxi University, Taiyuan 030006, China

**Keywords:** optical microfibre, optical nanofibre, fibre taper, optical sensor, tutorial

## Abstract

Micro/nanofibres (MNFs) are optical fibres with diameters close to or below the vacuum wavelength of visible or near-infrared light. Due to its wavelength- or sub-wavelength scale diameter and relatively large index contrast between the core and cladding, an MNF can offer engineerable waveguiding properties including optical confinement, fractional evanescent fields and surface intensity, which is very attractive to optical sensing on the micro and nanometer scale. In particular, the waveguided low-loss tightly confined large fractional evanescent fields, enabled by atomic level surface roughness and extraordinary geometric and material uniformity in a glass MNF, is one of its most prominent merits in realizing optical sensing with high sensitivity and great versatility. Meanwhile, the mesoporous matrix and small diameter of a polymer MNF, make it an excellent host fibre for functional materials for fast-response optical sensing. In this tutorial, we first introduce the basics of MNF optics and MNF optical sensors, and review the progress and current status of this field. Then, we discuss challenges and prospects of MNF sensors to some extent, with several clues for future studies. Finally, we conclude with a brief outlook for MNF optical sensors.

## 1. Introduction

The “ability” of using light to sense environmental changes or exotic objectives has been developed over more than a hundred million years during the evolution of creatures on this planet [1], while the “technique” of optical sensing was emerged only a few hundreds of years ago [2]. The great advance of the latter is the use of artificial devices to generate, propagate, convert or detect light in a precise and repeatable manner, among which engineering optical fields with certain confinement, like waveguiding light along dielectric wires proposed by Hondros and Debye in 1910, is of particular importance [3]. Historically, during the famous eclipse of 1919, when Eddington tested the theory of general relativity by observing the deflection of light by the Sun [4], light from a distant star was used to sense the gravitational bending of light, in which the “sample” to be measured was so large that no engineering on the probing light is required or possible. Compared with free-space probing light, artificially engineered optical fields are in increasing demand for detecting samples with much smaller sizes and/or weaker light-matter interactions. For example, for a non-absorptive sample with size below the wavelength of the probing light, and a free-space incoherent probing light with beam size much larger than the wavelength, it is difficult to separate the scattering signal from the background. To increase the signal to noise ratio, one of the most efficient approaches is increasing the optical confinement of the probing beam. The invention of highly coherent light source—“laser”—by Theodore Maiman in 1960 [5] drastically facilitated the use of confined optical fields, and the idea of guiding light in glass fibres proposed by Kao and Hockham in 1966 [6] greatly increased our ability to engineer optical fields with low loss, wavelength-scale confinement, high compactness, flexible redirection, and much larger light-matter interaction length, which opened a new field for optical sensing—fibre optical sensing—one of the most successful sensing technologies so far. While the fibre-optic technology have been well-established in the past 50 years [7], the growing demand for fibre optics with new dimensions and the rapid progress in related technology, have spurred great efforts for pushing forward fibre-based technology. It is clear that, with the increasing confinement of light from free-space propagation to guided modes in an optical fibre, the capacity of optical communication drastically increases. This is also true for enhancing the sensitivity of optical sensing, the resolution of optical imaging, and many other related technology. Usually, a better confinement of light yields a more powerful optical technology. 

**Figure 1:**
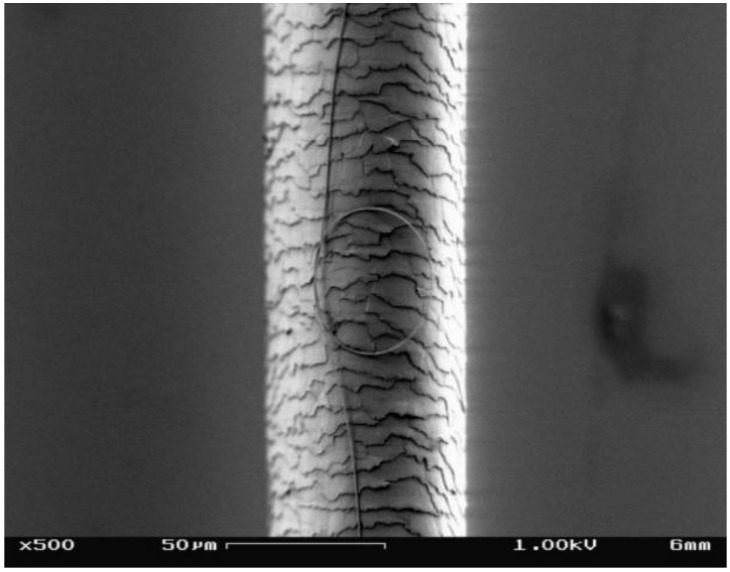
SEM image of a 500-nm-diameter silica MNF tied into a ring and placed on a 60-μm-diameter human hair.

As a combination of fibre optics and nanotechnology, mocro-/nanostructurization of optical fibres has been an important trend for exploring fibre-optic sensing technology on the micro or nanoscale [8,9,10,11,12,13,14,15,16,17,18,19,20,21]. It is obvious that, reducing the size of a sensing structure is usually an essential step to bestow the sensor with higher sensitivity, faster response, lower power consumption and better spatial resolution, and the optical micro/nanofibre (MNF) became one of the best candidates for this purpose. Fabricated by taper-drawing glass at high temperature or dissolved polymer at room temperature, an optical quality MNF can offer large length, extraordinary surface smoothness, high diameter uniformity and excellent mechanical flexibility, with diameter from tens of nanometers to micrometers (Figure 1) [22]. 

By using a micro/nanoscale solid cylinder as core and the outer surrounding space as cladding, the MNF is the simplest structure among all other micro/nanostructurized fibres or waveguides , in terms of fabrication, optical characterization, micro-manipulation, theoretical modeling and active functionalization [23,24].

Owing to its excellent geometric and material uniformity, wavelength or subwavelength diameter, high-index contrast between the solid fibre core and the surrounding medium (e.g., vacuum, gas or liquid), an MNF can guide light with low optical loss, outstanding mechanical flexibilities, tight optical confinement and large fractional evanescent fields [25], which makes it a versatile platform for optical sensing on micro/nanometer scale with special advantages including small footprint, fast response, high sensitivity, and low power consumption.

In recent years, rapid progress has been made in the field of MNF-based optical sensors, as have been summarized in a number of books and review articles [8,9,10,11,12,13,14,15,16,17,18,19,20,21]. In this tutorial, besides the basic introduction to MNF optics for optical sensors, we will review the up-to-date progresses in this field, and some of their closely related techniques. At the same time, we will discuss prospects and challenges of MNF optical sensors to some extent, with several clues for further studies. Finally, we will conclude with a brief outlook, and hope that some points in this paper can be helpful or realized in future studies. 

## 2. Optical MNFs

### 2.1. MNF Fabrication

**Figure 2:**
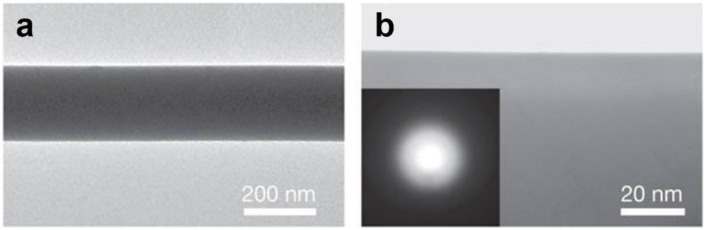
TEM images of (**a**) a 240-nm-diameter silica MNF and (**b**) the surface of a 330-nm-diameter silica MNF. The electron diffraction pattern (inset) demonstrates that the MNF is amorphous. Reprinted with permission from [22]. Copyright 2003 Springer Nature.

For non-absorptive optical micro/nanowaveguides, low surface roughness, high geometric and material uniformities are critical to achieve low waveguiding loss. Compared with many other micro/nanowaveguides fabricated by techniques such as electron-beam lithography and chemical growth, MNFs fabricated by taper-drawing glass fibre at high temperature offer much lower surface roughness, e.g., <0.2 nm RMS for silica MNFs (Figure 2), which is only determined by the capillary waves frozen on the surface during the cooling process [8]. 

**Figure 3:**
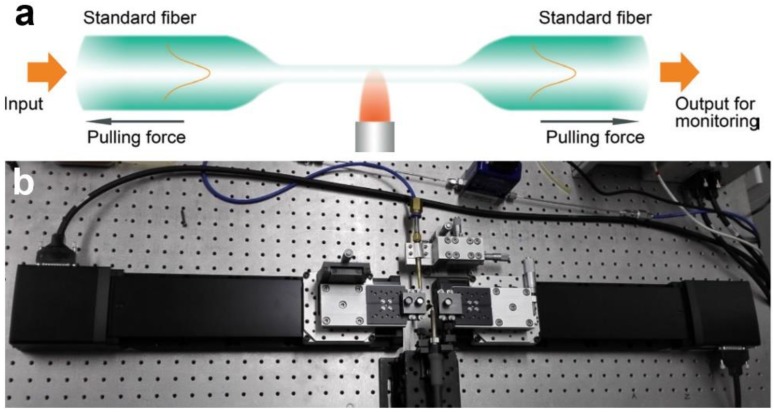
Taper-drawing of glass MNF. (**a**) schematic illustration of the drawing process; (**b**) A typical taper-drawing system for MNF fabrication.

A typical illustration of high-temperature taper-drawing process is shown in Figure 3a. A hydrogen flame is used to heat the fibre to its softening temperature. Under a certain pulling force, the fibre is stretched and elongated gradually with reduced diameter until the desired length or diameter is reached. Using this technique, the as-fabricated MNF is usually attached to the standard fibre through the tapering area at both ends, and is usually mentioned as a “biconical” fibre taper or MNF. Meanwhile, by measuring its optical transmission, it is possible to in-situ monitoring the waveguiding properties of the MNF during the pulling process in terms of propagation loss, multi-mode interference and group velocity delay [9,26,27]. Also, the amorphous structure and the surface tension of the molten glass bestows the MNF with perfect circular cross-section, which is ideal for obtaining waveguiding modes by analytically solving Maxwell’s Equations in a cylindrical coordinate [28].

Based on the taper-drawing process mentioned above, in recent years, a number of improvements on this technique have been reported for fabricating MNFs with various parameters including ultra-small diameters [29], reduced propagation losses [30,31,32], optimized tapering profiles and controllable cross-section geometries [33,34]. For example, recently, with an elaborately designed taper-drawing system (Figure 3b) with feedback from the in-situ transmission drop due to the cut-off of the high-order waveguiding modes, Xu et al. showed the possibility of drawing an MNF with precisely controlled diameter (deviation < 5 nm) [35]. 

**Figure 4:**
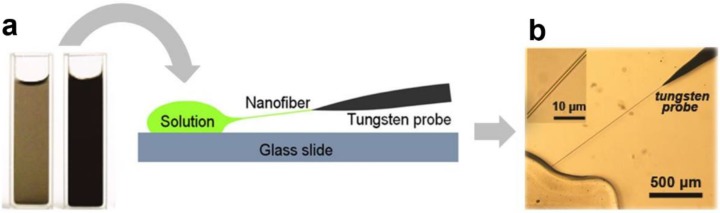
Taper-drawing fabrication of polymer MNFs from a dissolved polymer solution. (**a**) Schematic illustration of the drawing process; (**b**) Optical image of an as-drawn polymer MNF. Reprinted with permission from [37]. Copyright 2015 Springer Nature.

For polymer MNFs, a number of fabrication techniques have been successfully developed [24], among which physical drawing is the best approach to optical-quality MNFs with excellent geometric uniformity and surface smoothness. In a typical physical drawing fabrication, as shown in Figure 4, a sharp tip (e.g., a tungsten probe or a tapered fibre probe) is used to directly draw polymer MNFs out of a droplet of polymer solution at room temperature. By controlling the drawing speed (typically millimeters to meters per second, depending on the material and temperature) and the solution concentration, the diameter of the MNF can be roughly determined [36,37]. 

Besides the above-mentioned techniques, CO_2_ laser beams and electrical heaters have also been used for taper drawing glass or polymer MNFs, and glass MNFs have also been fabricated by local melting or chemical etching [38]. For more details of MNF fabrication, there have been a number of studies in recent literatures [8,9,10,11,12,13,14,15,16,17,18,19,20,38].

**Figure 5:**
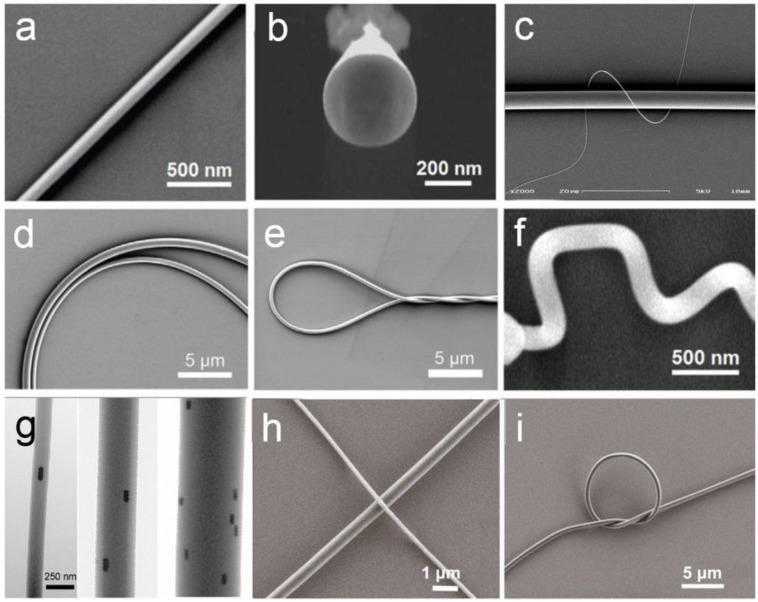
Electron microscope images of typical glass and polymer MNFs fabricated by physical drawing processes. (**a**) A 100-nm-diameter tellurite glass MNF. Reprinted with permission from [52]. Copyright 2006 OSA; (**b**) Cross section of a 400-nm-diameter tellurite glass MNF. Reprinted with permission from [52]. Copyright 2006 OSA; (**c**) A 160-nm-diameter silica MNF wrapped around a 4-μm-diameter silica MNF. Reprinted with permission from [29]. Copyright 2005 IOP; (**d**) Plastic bends of a 780- and a 490-nm-diameter silica MNFs; (**e**) A 300-nm-diameter silica MNF with a bending radius of 4 μm. Reprinted with permission from [29]. Copyright 2005 IOP; (**f**) 170-nm-diameter tellurite glass MNF with sharp plastic bends. Reprinted with permission from [52]. Copyright 2006 OSA; (**g**) Three PAM MNFs embedded with aligned Au nanorods. Reprinted with permission from [57]. Copyright 2012 ACS; (**h**) A 170- and a 510-nm-diameter PVA MNFs. Reprinted with permission from [37]. Copyright 2015 Springer Nature; (**i**) A 7-μm-diameter micro-knot tied with a 600-nm-diameter PVA MNF Reprinted with permission from [37]. Copyright 2015 Springer Nature.

To date, a variety of materials (e.g., silicate, phosphate, tellurite, fluoride, bismuth oxide and chalcogenide glasses, and a number of polymers) have been drawn into optical-quality MNFs. To bestow the as-fabricated MNFs with more functionalities, a number of post-fabrication techniques including micromanipulation [8,39], plastic bend [39], surface coating [40,41], embedding [42,43,44,45], index modulation [12], and fusion splicing [46,47,48,49,50,51] of MNFs have been reported. Moreover, a variety of novel structures, such as MNFs with active dopants [52], panda core to maintain polarization [53], elliptical cross-sections for high birefringence [34], or suspended cores to isolate surface contamination and contact leakage [54,55,56] have been also reported. For reference, Figure 5 shows electron microscope images of typical glass and polymer MNFs, in which the geometric uniformity and mechanical flexibility of MNFs are clearly seen. 

**Figure 6:**
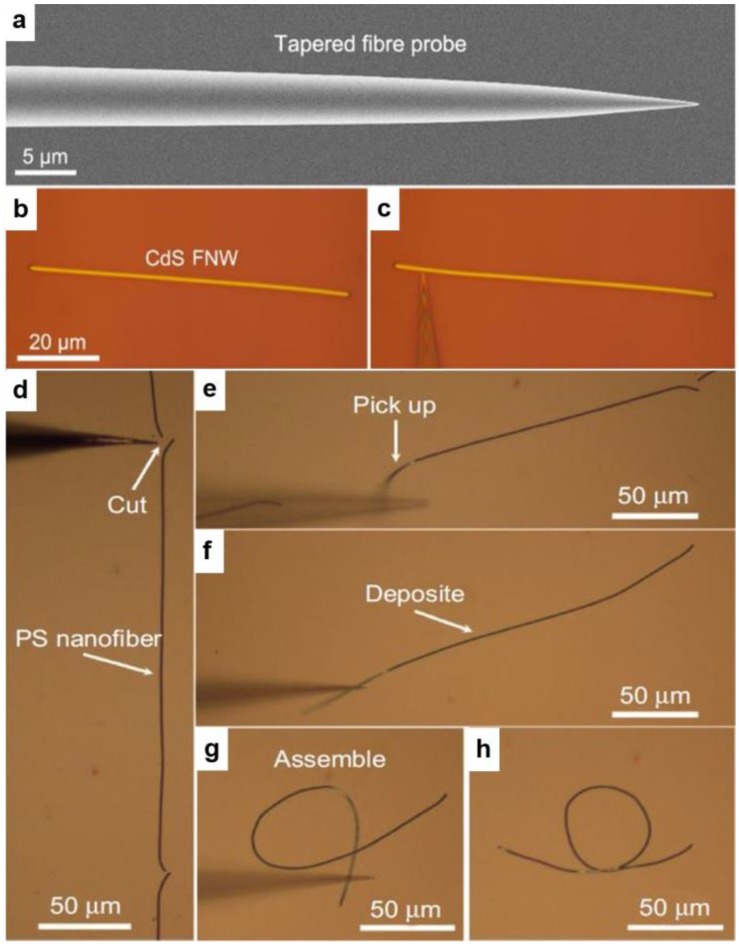
Micromanipulation of MNFs on a substrate. Reprinted with permission from [24]. Copyright 2013 Springer Nature.

Using precisely controlled tungsten or tapered fibre probes with tip sizes of tens to hundreds of nanometers (Figure 6a), MNFs placed on the surface of a certain substrate (Figure 6b, e.g., a silicon wafer) can be pushed (Figure 6c), cut (Figure 6d), picked up (Figure 6e), transferred and deposited (Figure 6f), bent into a loop (Figure 6g,h) under an optical microscope equipped with ultra-long working distance objectives. So far, a variety of MNF-based functional structures including microcouplers, resonators, interferometers, and loop mirrors have been experimentally realized [8,9,10,11], which have added new possibilities for MNF sensors, as discussed later.

### 2.2. Basic MNF Optics for Optical Sensing

#### 2.2.1. Propagation Constants

**Figure 7:**
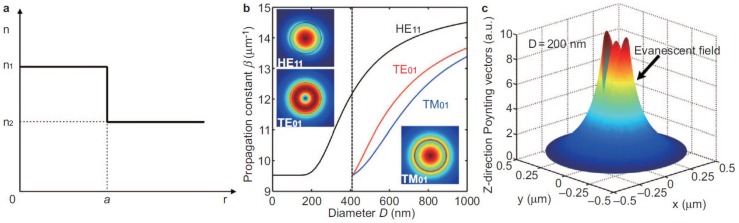
Mathematical modeling of an optical MNF. (**a**) Index profile of an MNF; (**b**) Numerical solutions of the propagation constant (*β*) of an air-clad PS MNF at 660-nm wavelength. Dotted line, critical diameter for single-mode operation. Insets, power distributions (Poynting vectors) of the three modes at the transverse crossplane of a 600-nm-diameter PS MNF; (**c**) Calculated Poynting vector of a 200-nm-diameter PS MNF guiding a 660-nm-wavelength light. Reprinted with permission from [24]. Copyright 2013 Springer Nature.

Because of the large index contrast (e.g., ~0.5) between the MNF core and the cladding and the wavelength-scale cross-section, the small index contrast (e.g., 0.05) in the glass-fibre preform that may remain after being drawn into a MNF, can be ignored in most cases. Thus, the MNF can be mathematically assumed to have a step-index profile (Figure 7a):

(1) $$n\left(r\right)=\left\{ \begin{array}{ll} n_{1}, & 0\;<\;r\;<\;a\\ n_{2}, & a\;\leq \;r\;<\;\infty \end{array}\right.$$

where the refractive indices (RIs) of the core and the cladding are assumed to be *n*_1_ and *n*_2_, respectively, and the core radius is *a*. 

For non-dissipative core materials, the propagation constant (*β*) of an MNF can be obtained by analytically solving the Helmholtz equations [25,28]:

(2) $$\begin{array}{l} \left(\nabla^{2}+n^{2}k^{2}-\beta^{2}\right)\vec{e}=0,\\ \left(\nabla^{2}+n^{2}k^{2}-\beta^{2}\right)\vec{h}=0 \end{array}$$

where *k* = 2*π*/*λ*, *λ* is the vacuum wavelength of the guided light.

Benefited from the perfect circular cross section of an MNF, Equation (2) can be analytically solved in cylindrical coordinate, with eigenvalue equations for waveguiding modes as follows for *HE_νm_* and *EH_νm_* modes:

(3) $$\left\{\frac{J_{\nu }^{\prime }\left(U\right)}{UJ_{\nu }\left(U\right)}+\frac{K_{\nu }^{\prime }\left(W\right)}{WK_{\nu }\left(W\right)}\right\}\left\{\frac{J_{\nu }^{\prime }\left(U\right)}{UJ_{\nu }\left(U\right)}+\frac{n_{2}^{2}K_{\nu }^{\prime }\left(W\right)}{n_{1}^{2}WK_{\nu }\left(W\right)}\right\}={\left(\frac{\nu \beta }{kn_{1}}\right)}^{2}{\left(\frac{V}{UW}\right)}^{4}$$

for *TE_0m_* modes:

(4) $$\frac{J_{1}\left(U\right)}{UJ_{0}\left(U\right)}+\frac{K_{1}\left(W\right)}{WK_{0}\left(W\right)}=0$$

and for *TM_0m_* modes:

(5) $$\frac{n_{1}^{2}J_{1}\left(U\right)}{UJ_{0}\left(U\right)}+\frac{n_{2}^{2}K_{1}\left(W\right)}{WK_{0}\left(W\right)}=0$$

where *J_ν_* is the Bessel function of the first kind, and *K_ν_* is the modified Bessel function of the second kind, and $U=a{\left(k_{0}^{2}n_{1}^{2}-\beta^{2}\right)}^{1/2}$, $W=a{\left(\beta^{2}-k_{0}^{2}n_{2}^{2}\right)}^{1/2}$, $V=k_{0}a{\left(n_{1}^{2}-n_{2}^{2}\right)}^{1/2}$.

By numerically solving Equations (3)–(5), waveguiding modes supported by the MNF can be obtained. For reference, Figure 7b shows *β* of the three lowest-order modes (*HE_11_*, *TE_01_*, *TM_01_*) of a polystyrene (PS) MNF (refractive index, RI ~1.59) waveguiding a 660-nm-wavelength light. It shows that, to operate a PS MNF in single mode in vacuum or air, the core diameter must be smaller than 410 nm. To show the spatial distribution of the waveguiding optical fields, calculated cross-sectional power distribution of a 600-nm-diameter PS MNF guiding the first three modes are given in insets of Figure 7b. The tight optical confinement is clearly seen, while considerably high surface intensity is also presented. The surface intensity and the fractional evanescent fields can be readily changed by the wavelength-to-diameter ratio. For reference, Figure 7c gives calculated Poynting vectors of a 200-nm-diameter PS MNF guiding a 660-nm-wavelength light, presenting abundant evanescent fields (>80% in power) confined and guided along the MNF, which is highly desired for high-sensitivity optical sensing within the vicility of the fibre surface. 

#### 2.2.2. Evanescent Coupling

**Figure 8:**
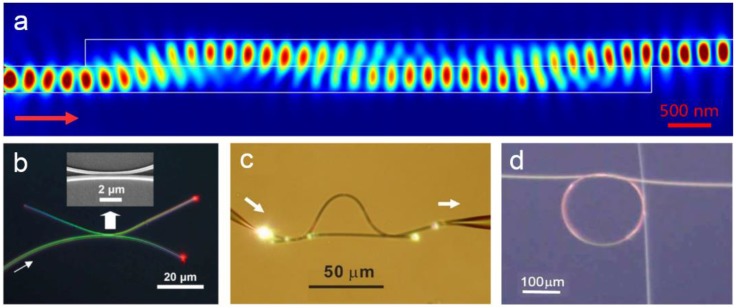
(**a**) FEM simulation of evanescent coupling of 633-nm light between two 350-nm-diameter silica MNFs; (**b**) Optical micrograph of an optical coupler assembled using two tellurite glass MNFs (350 and 450 nm in diameter respectively) on the surface of a silicate glass. The coupler splits the 633-nm-wavelength light equally. Reprinted with permission from [52]. Copyright 2006 OSA; (**c**) Optical micrograph of a MZI assembled with two 480-nm-diameter tellurite MNFs. Reprinted with permission from [59]. Copyright 2008 OSA; (**d**) Optical micrograph of a 200-μm-diameter microknot resonator add–drop filter assembled with two silica MNFs. Reprinted with permission from [60]. Copyright 2007 OSA.

Due to the large fractional evanescent fields of the MNF, evanescent coupling between MNFs or other 1-dimensional nanowaveguides can be very efficient, leading to high coupling efficiency within a short coupling length [58]. For reference, Figure 8a shows FEM simulation of evanescent coupling of 633-nm light between two 350-nm-diameter silica MNFs. The light is firstly propagated from left side to the right in the bottom MNF, when it encounters the upper MNF closely attached to the bottom one in parallel, almost all the energy is coupled into the upper MNF within a coupling length of merely 3 μm. The highly efficient coupling behavior makes it possible to assemble MNF-based branch couplers (Figure 8b), Mach-Zehnder interferometers (MZIs) (Figure 8c) or resonators (Figure 8d) with high compactness, which have been used as functional structures for optical sensing. 

#### 2.2.3. Bending Loss

**Figure 9:**
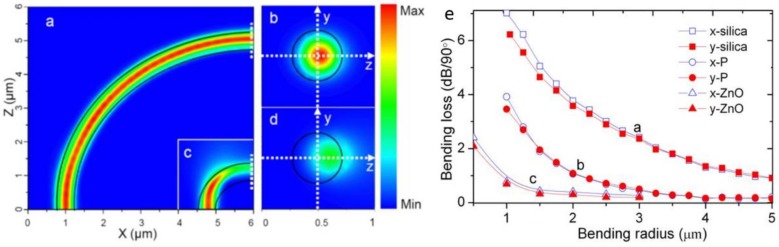
Electric field intensity distributions of (**a,b**) a 5-μm and (**c,d**) a 1-μm-bent silica MNFs. The wavelength of the quasi-*x* polarized light is 633 nm and the diameter of the MNF is 450 nm. (**e**) calculated bending loss of a 350-nm-diameter silica MNF, a 350-nm-diameter PS MNF, and a 270-nm-diameter ZnO nanowire at 633-nm wavelength (quasi-*x* and quasi-*y* polarizations), respectively. Reprinted with permission from [61]. Copyright 2009 OSA.

Owing to its high-index contrast, when the diameter-to-wavelength ratio is not very small (e.g., >0.7 for a silica MNF), an MNF with air cladding can guide light through sharp bends with low bending loss [61], which makes it possible to fabricate MNF-based ring resonators, MZIs and couplers with small footprints, and reduces the geometric sizes of sensors based on these functional structures. However, when the bend is really sharp, evident bending loss will appear. Figure 9a–d shows calculated electric field intensity distribution of guiding a 633-nm light through a 450-nm-diameter silica MNF with bending radius of 5 μm and 1 μm, respectively. While there is no obvious leakage in the 5-μm bend, the leakage of the 1-μm bend is clearly seen. For reference, Figure 9e gives calculated bending losses of a 350-nm-diameter silica MNF, a 350-nm-diameter PS MNF, and a 270-nm-diameter ZnO nanowire at 633-nm wavelength, respectively, clearly shown the increasing bending loss with decreasing bending radius and RI of the core material. On the other hand, the strong dependence of bending loss on the MNF diameter, bending radius, and core or environmental index, can also be used for sensing the change of environmental index, strain, curvature or force. 

#### 2.2.4. Adiabatic Taper

**Figure 10:**
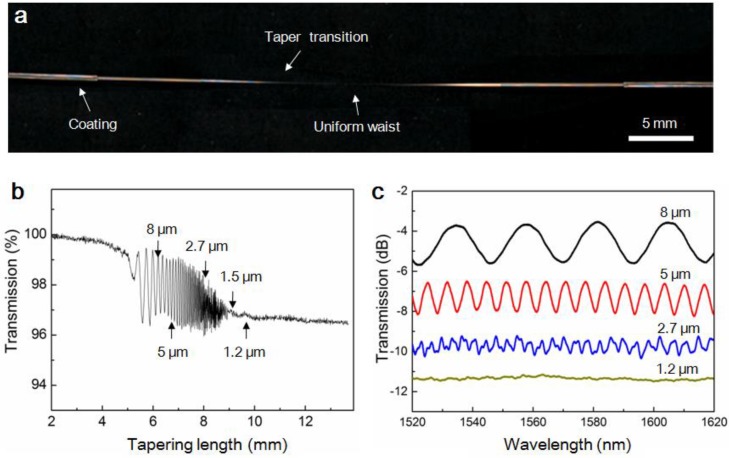
Optical characterization of silica MNFs tapered from a standard telecom single-mode fibre. (**a**) Optical micrograph of a 1.2-μm-diameter MNF tapered from a standard glass fibre; (**b**) In-situ transmittance measured during the tapering process at 1550 nm wavelength; (**c**) Transmission spectra of MNFs with different diameters (offset for clarity). Reprinted with permission from [62]. Copyright 2014 ACS.

For MNFs drawn from glass fibres, when the geometry of the taper is well controlled (usually with a small taper slope), the fundamental waveguiding mode in the glass fibre can be almost adiabatically converted into the fundamental waveguiding mode of the MNF [30,31]. As shown in Figure 10, with a relatively large taper length (Figure 10a), it is possible to maintain a transmission as high as 97% when the fibre is drawn into a subwavelength-diameter single-mode MNF (Figure 10b), where the single-mode cutoff diameter is about 1.2 μm for the 1.55-μm wavelength light. This high fibre-to-MNF efficiency is favorable for connecting the MNF with standard fibre system, as well as for reducing the optical power and the background noise in MNF-based optical sensors. In addition, the multimode interference observed in the MNF during the drawing process (Figure 10b,c), which is also sensitive to index change, have been used for MNF-based optical sensing.

## 3. MNF Optical Sensors

So far, there are a number of nice review articles on MNF optical sensors or similar devices [8,9,10,11,12,13,14,15,16,17,18,19,20,21]. To avoid much overlap, in this section, based on our previous review in 2014 [16], We will renew the progresses in this hot topic, with new development in this field. 

**Figure 11:**
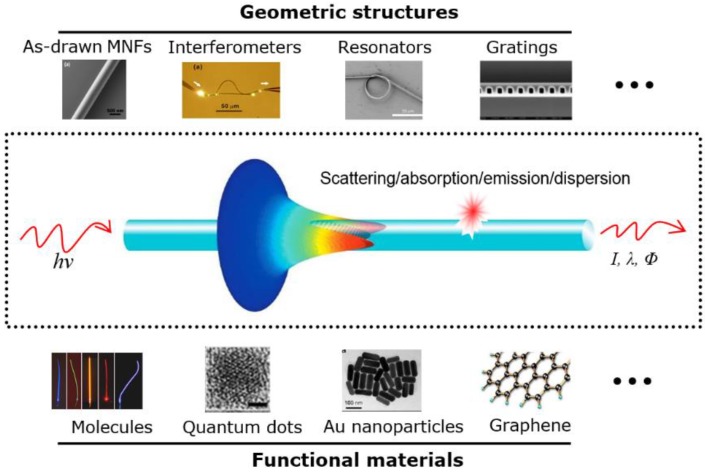
Schematic category of MNF-based optical sensors.

As introduced before, an MNF is distinguished from other 1-dimensional micro/nanowaveguides by its geometry and material, which can be phenomenally used for categorizing MNF-based sensors. As shown in Figure 11, the “geometric structures” employed for MNF sensors includes as-drawn MNFs, couplers, interferometers, resonators and gratings, while the “functional materials” includes molecules, nanoparticles, quantum dots, graphene layers and many other surface coatings. Also, because of the vast number of literatures in this fields, limited by the length, here we have to focus on MNFs with diameters typically less than 10 μm, and apologizes for the incompleteness of this brief review.

### 3.1. Geometric Structures

#### 3.1.1. As-draw MNFs

As-drawn MNFs, usually biconically connected to glass fibres or evanescently coupled to fibre tapers for coupling probing light in or collecting signal light out, are the most simple and straightforward structures for optical sensing. 

**Figure 12:**
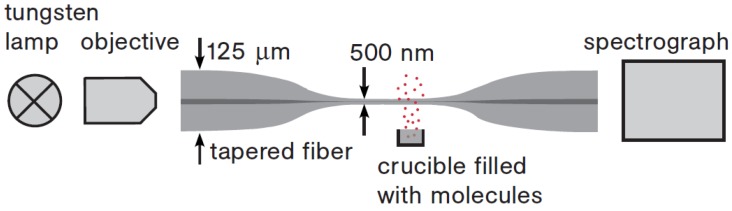
Scheme of the experimental set-up of an MNF optical sensor. White light from a tungsten lamp is transmitted through a tapered fibre with a 500-nm diameter MNF for measuring the absorbance of molecules deposited on the fibre surface with high sensitivity. Reprinted with permission from [63]. Copyright 2007 OSA.

When the MNF is used in gas or other low-index environment, in order to leave a considerably large fractional evanescent fields and generate signals large enough at its ouput, the diameter of the MNF is usually close to or below the wavelength of the probing light. For example, in 2007, relying on absorption of molecules adsorbed on the surface of a 500-nm-diameter silica MNF (Figure 12), Warken et al. reported an ultra-sensitive molecular sensor that was possible to detect sub-monolayers of 3,4,9,10-perylene-tetracarboxylicdianhydride (PTCDA) molecules by measuring spectral absorption around 500-nm wavelength [63]. 

**Figure 13:**
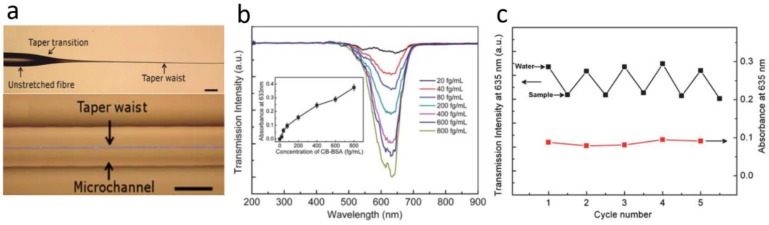
Microfluidic chip integrated MNF optical sensors. (**a**) Optical micrograph of a biconical MNF before (up) and after (bottom) integrated into a microfluidic channel; (**b**) Transmission spectra of different CB-BSA concentrations for the 900 nm diameter MNF. Inset: absorbance at 633 nm wavelength versus BSA concentrations; (**c**) Cycling measurement with 500 pM MB solutions for a 900-nm-diameter MNF. Reprinted with permission from [64]. Copyright 2011 RSC.

For MNFs used in a liquid or a matrix having an index much higher than the air (but still lower than that of the MNF core), which is a common case in biochemical applications, the diameter of the MNF can be larger. For example, by integrating a 900-nm-diameter silica MNF into a microfluidic chip (Figure 13a), and using a broadband white light as probing light to measure the absorbance of bovine serum albumin around 630-nm wavelength, Zhang et al. obtained a detection limit down to 10 fg mL^−1^ (Figure 13b) [64]. Also, the excellent reversibility of using the MNF sensor for biochemical sensing is observed in measuring methylene blue solution when the concentration is decreased to 500 pM level (Figure 13c). 

**Figure 14:**
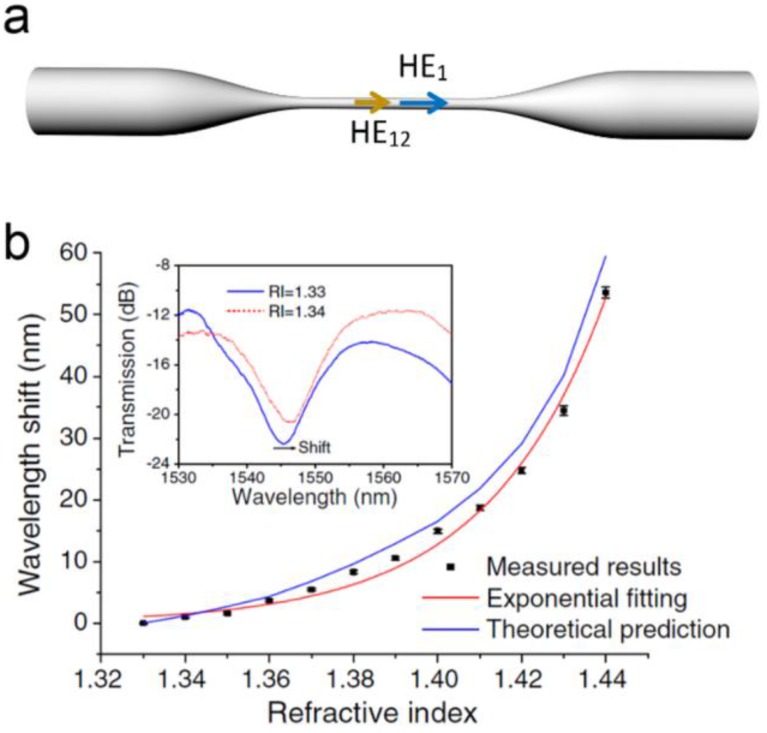
MNF optical sensors based on multimode interference. (**a**) Schematic illustration of two modes co-propagation in an MNF; (**b**) Calculated and measured peak wavelength shifts as a function of the RI in a multimode MNF. Inset, measured spectral responses of a MNF with indices of surrounding liquids of 1.33 and 1.34, respectively. Reprinted with permission from [66]. Copyright 2011 OSA.

With diameter larger than the wavelength, a biconical MNF can be operated in multimode, in which the multimode interference can be used for optical sensing [65]. For example, Figure 14a illustrates the co-propagation of *HE_11_* and *HE_12_* modes in an MNF for interference. The relative phase difference (*Δϕ*) of the two modes can be obtained as *Δϕ = Δβ·l*, where *Δβ* is the difference in propagation constants of the two modes, and *l* is the interaction length. When *Δβ* and/or *l* is changed due to the environmental change (e.g., RI, temperature or strain), the relative phase change *Δϕ* will induce spectral shift at the output, leading to a loss-independent MNF sensor. For example, in 2011, based on the multimode interference of a 1.55-μm-wavelength probing light guided through a 30-μm-diameter silica biconical MNF, Wang et al. experimentally demonstrated a maximum sensitivity of 1913 nm/RIU (Figure 14b) [66], resulting in a resolvable index change of 5.23 × 10^−6^ for a resolvable wavelength change of 0.01 nm, which was among the highest resolutions reported in fibre optical RI sensors. 

Recently, based on the multimode interference, a number of MNF optical sensors have been report for measuring RI [67,68], magnetic fields [69], strain [70,71] and temperature [72]. Meanwhile, by exploring the Faraday-effect induced polarization rotation [73], pressure [74] or strain [75] induced optical path changes of guided modes of biconical MNFs, MNF sensors for current, acceleration and acoustic waves have also been reported.

#### 3.1.2. Directional Couplers

**Figure 15:**
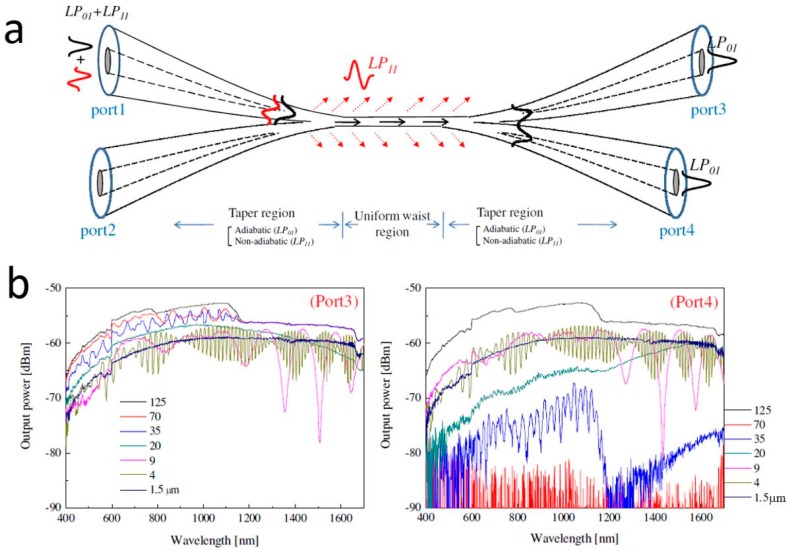
Single-mode biconical MNF coupler at telecommunication band. (**a**) Schematic diagram; (**b**) Spectral outputs of the MNF directional coupler. Reprinted with permission from [76]. Copyright 2009 OSA.

A directional coupler is a highly effective structure for optical sensing when the coupling efficiency is strongly dependent on the index change of fibre core or its surrounding medium. In 2009, Jung et al. reported a single-mode biconical MNF coupler at telecommunication band [76]. Relying on a higher-order filtering characteristics of a subwavelength diameter MNF (a 1.5-μm-diameter MNF operated at 1.55-μm wavelength) [77], they realized broadband (400~1700 nm) single-mode operation of a 2 × 2 fused MNF coupler [Figure 15a], which effectively suppressed higher-order modes presented at the input fibre and provided efficient power splitting into the fundamental mode at the two output ports [Figure 15b], showing the possibility for optical sensing based on the coupling-efficiency-dependent response. So far, MNF directional couplers have been employed for optical sensing a variety of measurands such as force [78], magnetic fields [79], current [80], seawater salinity [81] and temperature [81,82]. 

#### 3.1.3. MZIs

MZI is one of the mostly used structures for optical sensing. When one arm of an MZI is replaced by an MNF guiding high fractional evanescent fields, it is possible to generate larger optical path difference compared with other optical waveguides. Therefore, incorporating optical MNFs into MZIs for phase-sensitive optical measurement may offer high sensitivity with miniature sensor size.

**Figure 16:**
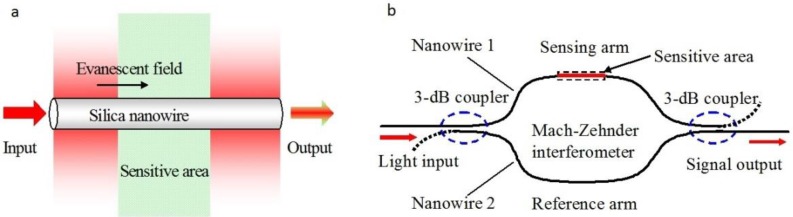
Schematic diagram of (**a**) a silica MNF sensing element and (**b**) an MNF sensor with a Mach-Zehnder interferometer. Reprinted with permission from [83]. Copyright 2005 OSA.

**Figure 17:**
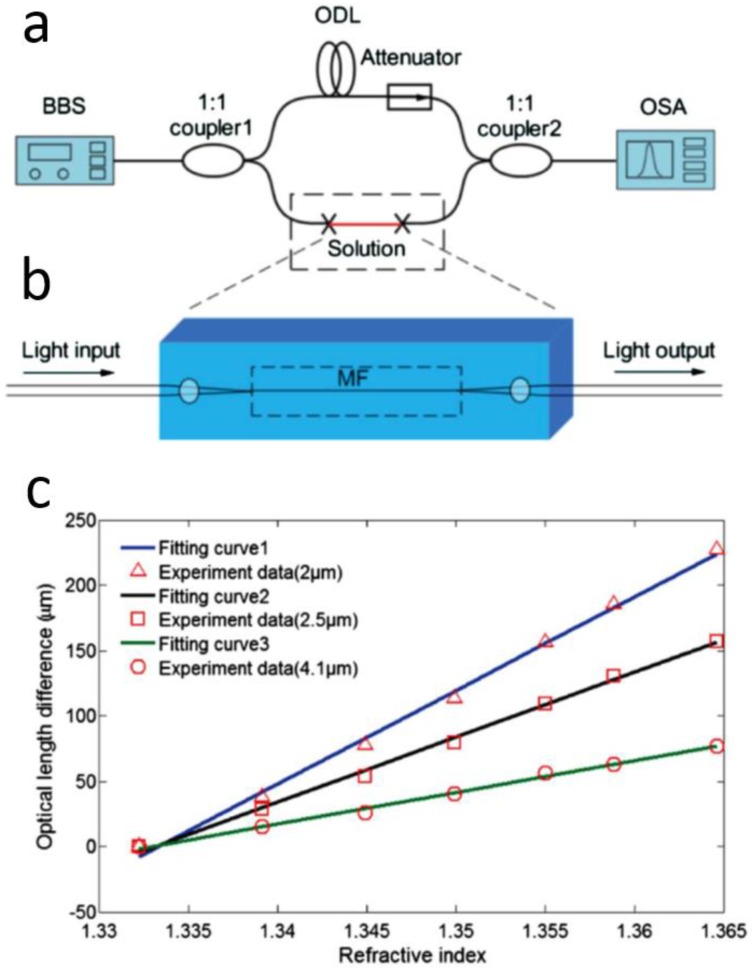
MNF-based MZI RI sensor. (**a**) Schematic configuration; (**b**) Schematic diagram of the sensing arm. BBS, broadband light source; ODL, optical delay line; OSA, optical spectrum analyzer; MF, microfibre; (**c**) Optical length variation with the RI at different MNF diameters. Reprinted with permission from [84]. Copyright 2012 OSA.

In 2005, based on numerical simulation of an MZI assembled with subwavelength-diameter MNFs (Figure 16), Lou et al. predicted that, for RI measurement in liquid environment, such an MNF MZI can provide a sensitivity one order of magnitude higher than those of conventional waveguide MZIs [83]. So far, a number of MNF-based MZIs have been employed for phase-sensitive optical sensing [84,85,86,87,88,89,90]. For example, in 2012, Wo et al. reported a simple and robust RI sensor based on an MNF MZI (Figure 17) [84]. By using a 2-μm-diameter silica MNF as the sensing arm, and using a tunable optical delay line to compensate the change of the optical length difference, they obtained a RI sensitivity as high as 7159 μm/RIU. Jasim et al. reported a MNF-MZI-based current sensor, with a slope efficiency of 60.17 pm/A^2^ [85]. In 2013, by integrating a Ag nanowire with an MNF MZI, Li et al. demonstrated a hybrid photon-plasmon MZI for fibre-compatible plasmonic sensing, with a response time of 0.3 s and a sensitivity better than 100 ppm for NH_3_ gas sensing [86]. In 2015, Luo et al. reported that, near the dispersion turning point of the multimode MNF-based MZI, it is possible to achieve very high RI sensitivity (e.g., 10777.8 nm/RIU) [87].

#### 3.1.4. MNF Gratings

Optical fibre gratings, especially fibre Bragg gratings (FBG), is one of the most successful fibre-based structures for optical sensing [91]. Owing to its high compactness, strong near-field interaction with the surrounding medium, and high resistance to mechanical and thermal shocks, MNF gratings may offer special advantages in optical sensing including high sensitivity, small footprint, large dynamic range and fast response, and have been attracting increasing interest in recent years [12].

**Figure 18:**
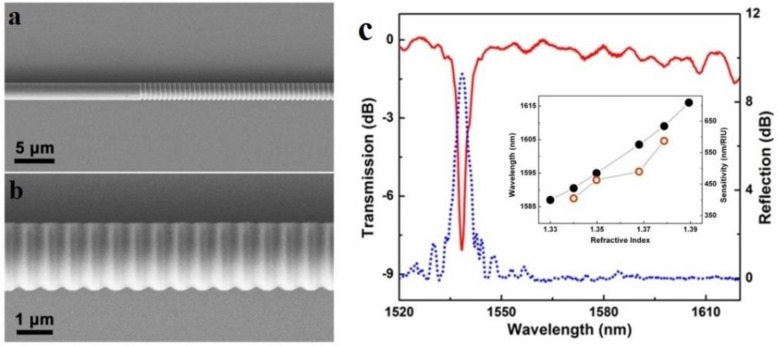
MNFBG fabricated by FIB. (**a**) SEM image of a MNFBG inscribed on a 1.8-μm-diameter silica MNF; (**b**) Transmission and reflection spectra of the MNFBG. Inset, dependence of the reflection wavelength shift on the ambient RI (black dot line) and the corresponding RI sensitivity (red hollow dot line) of the MNFBG used for measuring the RI of a glycerin solution. Reprinted with permission from [95]. Copyright 2011 OSA.

In principle, an MNF Bragg gratings (MNFBG) is a kind of a fibre Bragg gratings (FBG) with much smaller fibre diameter and shorter overall length. However, within the much shorter length, to obtain an evident grating effect, the index contrast of the MNF gratings has to be much higher than that in a conventional FBG. In 2005, Liang et al. fabricated an MNFBG by chemically etching a 6-μm-diameter silica MNF, and successfully used it for optical index sensing in liquids [92]. In 2010, Fang et al. reported MNFBGs fabricated by femtosecond laser pulse irradiation [93]. Using a 2-μm-diameter silica MNF gratings, they reported a maximum sensitivity of 231.4 nm/RIU at a RI of 1.44. Around the same time, Zhang et al. reported an MNFBG written in a photosensitive MNF using KrF excimer laser, and demonstrated a sensitivity of 102 nm/RIU (at a RI value of 1.378) in a 6-μm-diameter MNF [94]. In 2011, using focused ion beam (FIB) to milling the sidewall of a microfibre, Liu et al. demonstrated a 518-μm-length 1.8-μm-diameter MNFBG and a sensitivity of 660 nm/RIU for RI measurement around 1550 nm wavelength (Figure 18) [95]. Around the same time, Kou et al. reported a MNFBG fabricated by FIB milling, and demonstrated temperature sensing from room temperature to around 500 °C with a sensitivity of about 20 pm/°C near the resonant wavelength of 1550 nm [96]. It is also found that, an MNFBG with smaller MNF diameter and higher order mode may offer higher RI sensitivity [97]. 

Besides the above-mentioned MNFBGs, a variety of designs including long period gratings (LPG) [98,99,100,101], evanescently coupled gratings [102,103], Type IIa Bragg gratings [104], and chirped Bragg gratings [105], have also been fabricated on MNFs and used for high-sensitivity optical sensing of measurands from RI, force to immunity [98,99,100,101,102,103,104,105,106,107]. 

#### 3.1.5. MNF Resonators

**Figure 19:**
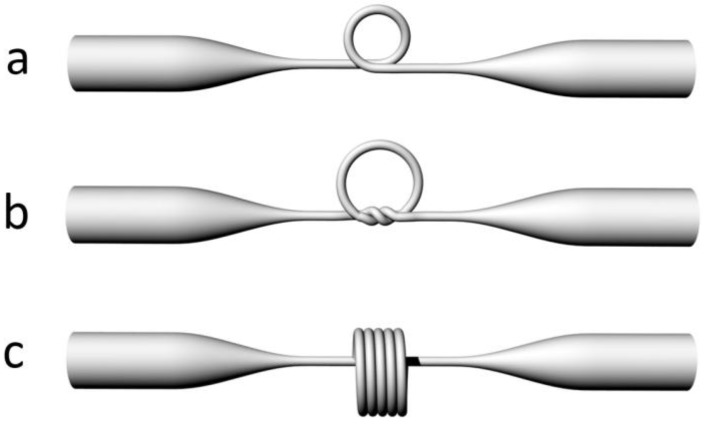
Schematic diagram of typical MNF-based optical resonators in forms of (**a**) a loop; (**b**) a knot; and (**c**) a stack of coils.

When an MNF is assembled into a closed loop, a ring resonator can be formed by evanescent coupling at the overlapping area. Typical geometries of this kind of resonator are schematically illustrated in Figure 19, in which the geometries of a loosely assembled loop (a), a tied knot (b), and a stacked multicoil (c) are schematically illustrated. Depends on the ring size and geometry, typical Q-factor of an MNF resonator varies from 10^2^ to 10^6^ [8]. 

As the simplest structure in MNF resonators, the MNF loop resonator has been intensively investigated. In 2006, Sumetsky et al. reported an MNF loop resonator with an intrinsic Q-factor of 630,000, and successfully used it for ultrafast temperature sensing [108]. Benefitted from the high Q-factor and the miniaturized structure, the sensor offered a temperature resolution down to 0.1 mK, and a response time as fast as microseconds. 

While the MNF loop is simple and high-Q, the loop structure maintained by the van der Waals and electrostatic forces is difficult to operate with high mechanical stability, especially in liquids. To enhance the robustness, in 2007, Guo et al. reported a copper-rod-supported loop resonator assembled by wrapping a 2.8-μm-diameter MNF around a 460-μm-diameter copper rod [109,110], under critical coupling condition, the MNF loop showed a sensitivity of 1.8×10^−5^ for refractive-index measurement. Embedding a free-standing MNF loop inside a low-index substrate (e.g., a polymer matrix) is another possible route to fabricate a robust micro-resonator for sensing applications [111,112,113], although the substrate may reduce the sensitivity of the sensor.

**Figure 20:**
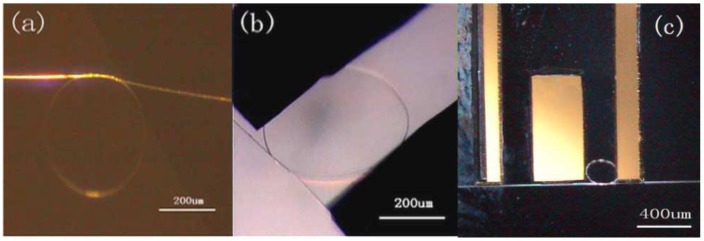
Optical accelerometer based on an MNF knot resonator. (**a**) Optical micrograph of the MNF knot with a diameter of 386 μm, which is assembled with a 1.1-μm-diameter silica MNF; (**b**) Surface profiler picture of the accelerometer; (**c**) Optical micrograph of the accelerometer. Reprinted with permission from [115]. Copyright 2009 IEEE.

To enhance the mechanical stability of free-standing MNF ring resonators without substrates, in 2006, Jiang et al. tied a free-standing MNF into a knot [114], in which the knot structure was maintained by the friction of the microfibre at the joint area under the tension of the elastically bent knot, and was proved highly stable in water with Q factors up to 31,000 and finesse of 13. Based on MNF knot resonators, a variety of sensing structures have been reported [115,116,117,118,119,120,121,122,123,124]. For example, in 2009, Wu et al. demonstrated a micro-electromechanical system (MEMS) based optical accelerometer combined with a 386-μm-diameter knot resonator fabricated by a 1.1-μm-diameter silica MNF [115]. As shown in Figure 20, the MNF knot had a Q-factor of 8500 and was used for vibration measurement of the MEMS structure. The experimental results showed that the MNF accelerometer had a sensitivity of 654.7 mV/g, with a dynamic range of 20 g. In 2011, using a copper-wire-wrapped MNF knot resonator, Lim et al. demonstrated tuning the resonant wavelength of the MNF resonator by applying electric current to the copper wire [118], and realized a compact current sensor with the maximum tuning slope of 51.3 pm/A^2^. Besides the above-mentioned examples, in recent years, MNF-knot-based optical sensors [125,126,127,128,129,130,131,132,133,134,135] have been widely explored for measurement of RI, humidity, temperature, and magnetic field. 

Firstly demonstrated by Sumetsky in 2004 [136], the three-dimensional multicoil structure is suggested to have a potential to reach ultrahigh Q factors [108]. In 2007, Xu et al. calculated a refractometric sensor based on a coated MNF coil resonator, and predicted a sensitivity up to 700 nm/RIU [137]. Shortly after, the same group experimentally assembled a 5-turn Teflon-coated 3D MNF coil with a 50 mm length 2.5 μm diameter silica MNF, and demonstrated a sensitivity of about 40 nm/RIU for index sensing of mixtures of isopropyl and methanol [138]. Since then, a number of MNF coil sensors have been reported for optical sensing of absorption [139], temperature [140], and current [141]. 

Besides the circular geometries mentioned above, recently, there are a number of novel MNF-based resonator structures, such as MNF-gratings-based cavities [142,143,144], microsphere/particle-attached WGM cavities [145,146] and theta-shaped structures [147], which we will not go into details here.

### 3.2. Functional Materials

#### 3.2.1. Functional Dopants or Inclusions

**Figure 21:**
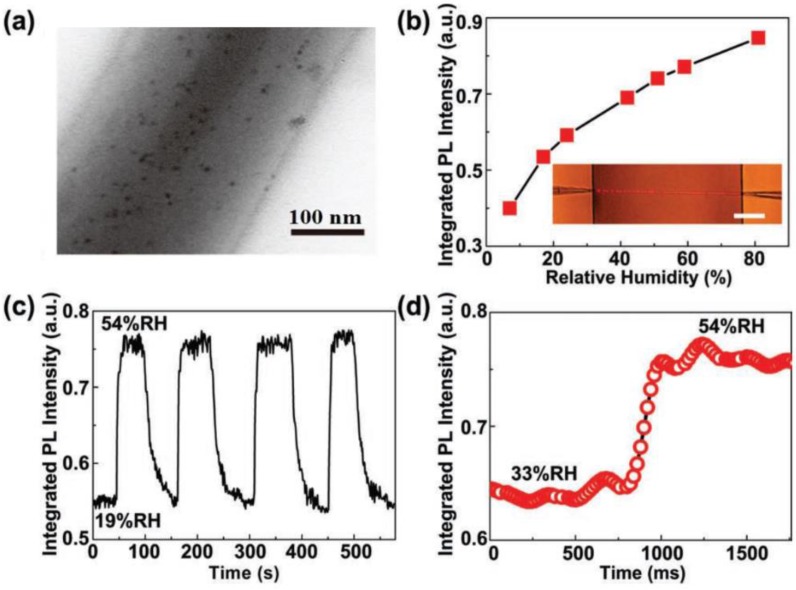
Quantum-dot activated polymer MNF optical sensor. (**a**) TEM image of a 280-nm-diameter PS MNF embedded with CdSe quantum dots; (**b**) PL intensity of the MNF exposed to ambient relative humidity (RH) ranging from 7% to 81%. Inset, optical micrograph of the sensing element. Scale bar: 50 μm; (**c**) Response of the MNF sensor to alternately cycled 54% and 19% RH air; (**d**) Temporary response of PL intensity to the sudden change of humidity. Reprinted with permission from [153]. Copyright 2011 WILEY-VCH.

**Figure 22:**
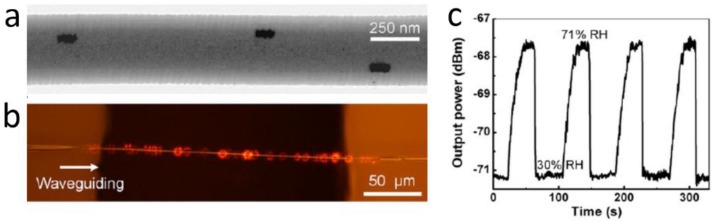
Au-nanorod activated polymer MNF optical sensor. (**a**) TEM image of a 540-nm-diameter PAM MNF embedded with Au nanorods; (**b**) Optical micrograph of a 785-nm-wavelength light waveguided along the 540-nm-diameter MNF; (**c**) Reversible response of the MNF output tested by cycling between 30 and 71% RH air. Reprinted with permission from [57]. Copyright 2012 ACS.

In addition to geometric structures, an MNF can also be functionalized by adding functional materials into the fibre core. Unlike in optical fibres for telecommunications and lasers, in which rare-earth dopants can be added into molten glass at high temperature, here the functional materials for optical sensing, e.g., organic molecules, typically cannot suffer the high temperature of the molten glass. Therefore, polymer MNFs drawn from room temperature solutions become the optimum substrates to host the functional materials. Generally, polymer MNFs are excellent hosts for exotic dopants or inclusions, including dye molecules [148,149,150,151], chemical indicators [36,152], quantum dots [153,154,155] and metal particles [57,156]. In 2008, based on spectral response of an acidic-to-basic form change of bromothymol blue (BTB) mixed in a 270 nm diameter poly(methyl methacrylate) (PMMA) MNF, Gu et al. demonstrated an MNF ammonia sensor with a detection limit of 3 ppm and a response time of about 1.8 s, much faster than conventional ammonia sensors [36]. In 2010, Meng et al. fabricated high-quality PS MNF with low-concentration CdSe/ZnS QD inclusions (Figure 21a) [153]. Based on the surface passivation of QD emission in a 480-nm-diameter MNF, a miniaturized optical humidity sensor with fast response (~90 ms) and ultra-low optical power (~100 pW) was successfully demonstrated (Figure 21). Metal nanoparticles have also been used in MNFs for plasmonic sensing. In 2013, by embedding a 540-nm-diameter PAM MNF with Au nanorods whose localized surface plasmonic resonance frequency was strongly dependent on environmental RI, Wang et al. demonstrated a low-power fast-response optical humidity sensor that is intrinsically immune to photobleaching (Figure 22) [57]. Recently, more functional materials, such as photochromic dyes [157], proteins [158] and ZnO nanostructures [159], have been incorporated with MNFs for optical sensing. 

#### 3.2.2. Functional Coatings

To functionalize glass MNFs with exotic materials, one of the most convenient approaches is to put them on the fibre surface. In 2005, Villatoro et al. coated a 1.3 μm diameter silica MNF with a 4-nm-thickness palladium film [40]. Relying on its hydrogen-concentration-dependent transmission at 1550 nm wavelength, they successfully operated the coated MNF as a fast-response (~10 s) miniature hydrogen sensor with low detection limit. In 2008, by coating a 680 nm diameter MNF with a 80 nm thickness gelatin layer whose RI changed with environmental humidity, Zhang et al. demonstrated an MNF optical sensor operated within a wide humidity range (9–94% RH) with high sensitivity, good reversibility and a 70-ms response time [41]. In 2010, by decorating a 10-μm-diameter silica MNF with PdAu nanoparticles on surface, Monzon-Hernandez et al. demonstrated a silica MNF hydrogen sensor [160] that was capable of detecting low concentrations of hydrogen (up to 8%) at room temperature with response times on the order of seconds, much faster than many other hydrogen sensors that exploit phenomena at the nanoscale. 

**Figure 23:**
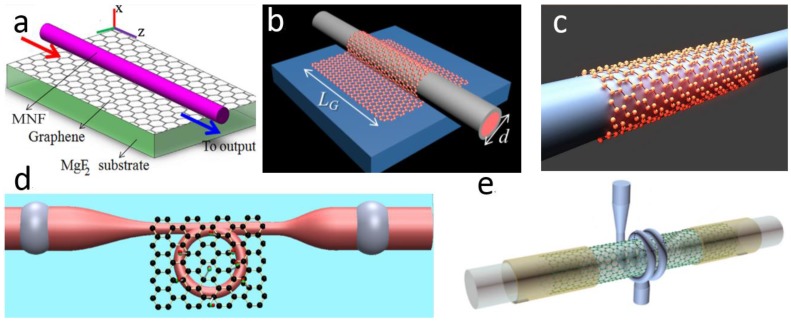
Typical configurations of hybrid graphene-MNF structures. The graphene layer is placed (**a**) beneath, Reprinted with permission from [163]. Copyright 2013 OSA; or (**b**) on the top of a substrate-supported MNF, Reprinted with permission from [164]. Copyright 2014 OSA; (**c**) wrapped around a free-standing MNF, Reprinted with permission from [62]. Copyright 2014 ACS; or incorporated with (**d**) an MNF ring, Reprinted with permission from [167]. Copyright 2016 OSA; and (**e**) an MNF coil. Reproduced from [168], with the permission of AIP Publishing.

More recently, using graphene atomic layers to detect adsorbed gas molecules has been attracting much attention due to its high sensitivity and low detection limit [161]. Graphene-activated MNFs become a new functional structure for optical sensing, with typical configurations shown in Figure 23. The graphene layer can be placed either beneath (Figure 23a) or on the top (Figure 23b) of a substrate-supported MNF, wrapped around a free-standing MNF (Figure 23c), or incorporated with a MNF ring (Figure 23d) and coil (Figure 23e). In 2012, based on index change when adsorbing molecules on the graphene surface, Yao et al. proposed to integrates the graphene film with a silica MNF to detect the molecular concentration based on intensity measurement of the TE modes [162]. In the following years, the same group have experimentally developed a series of hybrid graphene-MNF structures for gas sensing [163,164,165,166,167]. For example, in 2014, by using a graphene-coated MNF multimode interferometer for gas sensing, Yao et al. realized sensitivities up to ~0.1 ppm for NH_3_ gas detection and ~0.2 ppm for H_2_O vapor detection [164]. In 2016, based on a graphene oxide coated MNF knot resonator, Yu et al. demonstrated a sensitivities of ~0.35 pm/ppm for NH_3_ and ~0.17 pm/ppm for CO detection [167] Meanwhile, using hybrid graphene-MNF structures for current [168], temperature [169] UV light [170] and DNA [171] sensing have also been reported by other groups. 

In addition, many other functional coating materials, such as rare-earth ions [172], nanoporous polyelectrolyte [173] and propylene carbonate [174] have been successfully incorporated with MNFs, which has greatly enhanced the versatilities of MNFs in optical sensing applications.

## 4. Challenges and Prospects 

So far, a variety of MNF optical sensors have been demonstrated for physical, chemical, and biological applications. Rapid progresses on MNFs with new functional structures and materials, as well as new mechanism or effects for optical sensing, will continue to bestow MNF-based optical sensors with new opportunities. To take a glimpse into the future, I will try to discuss the challenges and prospects of this field, based on a revisit of optical properties of MNFs. 

As mentioned before, one outstanding advantage of a MNF is its ability to low-loss waveguiding light with tightly confined large fractional evanescent fields (e.g., a subwavelength confinement with more than 20% fractional power in evanescent fields), which is enabled by its atomic level surface roughness and extraordinary diameter uniformity, and is only possible in a glass MNF. For particle-like samples (e.g., protein and many other bio-molecules), confining the probing field to a size comparable to that of the sample, and catching the sample in the optical near field, will greatly enhance the light-sample interaction and consequently the sensitivity of the sensor [175,176]. For smaller particles, it is always possible to use higher-index glass and/or shorter-wavelength light to increase the optical confinement while maintain the same level fractional evanescent fields, which may lead to MNF sensors with extremely high sensitivity, e.g., single-small-molecule level, when incorporated with other functional structures. 

Meanwhile, qualitatively, when we discussing the sensitivity of a glass MNF, we may consider the MNF as an unfolded glass microsphere cavity, as both have atomic-level surface roughness and stable modal fields. Under optimized conditions, the both structures may share some similarities in optical sensing in terms of sensitivity, detection limit and signal processing, which may offer cross-reference for the both structure for further improvement. 

Ultimately, one may ask “how sensitive can an optical MNF be?” As a kind of measurement, generally, it should be determined by the Heisenberg’s uncertainty principle [177], seemingly distant for MNF sensors at this stage? However, some clues have been shown by researchers in atom optics. For example, in 2006, Le Kien et al. theoretically investigated the scattering of an evanescent field by a single cesium atom outside a subwavelength diameter silica MNF [178], and showed that in the close vicinity of the fibre surface, the ratio of the optical power scattered by a single atom could reach ~40%, corresponding to a kind of “sensitivity” better than one atom. Of course, here the probing light is assumed to be monochromatic with wavelength corresponding exactly to the atomic transition, and the atom is extremely “cold”. With this single-atom sensitivity in mind, and compare it with the up-to-date performance of the MNF sensors, the future challenge for an ultra-sensitivity MNF sensor is to find a balance point between—compromising one to enhance another—depending on the sample and the available conditions such as probing light, fabrication precision, photodetection and allowed time for the measurement. 

Another noticeable merit of the MNF is its ultra-low stiffness and high mechanical flexibility. For example, with the same Young’s modulus, the force required to bending a 400-nm-diameter silica MNF into a certain shape is about 100,000 times smaller than a standard silica fibre. Meanwhile, at room temperature, the maximum tolerable strain in a silica MNF is much larger than in standard silica fibre, as has been indicated by the much higher tensile strength of the MNF [22,179]. These mechanical properties are favorable for optical sensing in micro/nano-electromechanical systems, as well as in kinetic micro-biosystems. At the same time, an MNF also has a very low mass or inertia, e.g., a 200-nm-diameter 10-μm-length silica MNF is about 10^−15^ kg in mass or 10 fN in weight, which is comparable to the photon momentum of a 10-μW-power light, making it highly optomechnically sensitive. This ultralow inertia, when incorporated with tight confinement of both photons and phonons in the same MNF, may lead to strong optoacoustic effects [180], and add new dimension for MNF-based sensing of physical, mechanical or material properties of micro/nanoscale structures. 

Again, benefitted from the tightly confined large fractional evanescent fields, together with optimized taper shape for minimizing the overall length [181], compared with many other waveguides, an MNF can achieve the similar sensitivity with much shorter interaction length, or handle samples with much smaller quantity. For example, in 2015, by waveguiding a 473-nm-wavelength probing light along a 800-nm-diameter MNF crossing a 5-μm-wide microfluidic channel, Zhang et al. demonstrated an MNF optical sensor with sample requirement down to 1 femtoliter [182]. MNF sensors optimized in this way may find potentials in trace detection of chemical or biological samples. 

In addition, in recent years, as useful tools, optical MNFs have attracted increasing interest in atom physics. A variety of MNF-based techniques have been developed for trapping, coupling, manipulating and measuring cold atoms [14,178,183,184,185]. These MNF-coupled atom systems, could be highly sensitive to exotic disturbance, which may open new opportunities for MNF-based optical sensors, not only for measuring the atom ensembles inside [186], but also for sensing world outside. 

More challenges may come from the practical applications. Firstly, as an open sensitive structure relying on evanescent fields in the vicinity of the surface, protecting the MNF from surface contamination is usually required. Although a number of approaches, including integrated with microfluidic channels [64,182,187], suspended in capillary tubes [188], embedded in low-index polymers [42,43,44,45] have been successfully developed for packaging MNF-based sensors in recent years, the challenge remains in many cases since extremely high cleanness is typically required for high-sensitivity sensors, especially when they are designed for long-term operation. Fortunately, the structural and optical properties of glass MNFs are usually very stable when they are properly packaged, and the package is largely an issue of conventional techniques, although making it cost-effective is another challenge. 

Secondly, unlike standard optical fibres that can be routinely manufactured with high precision, repeatability and yield, drawing MNFs with high precision and repeatability remains challenging, although great progresses have been made in this subject in recent years.

## 5. Conclusions

So far we have introduced the principles and applications of MNF-based optical sensors, reviewed the up-to-date progresses, and discussed prospects and challenges of MNF optical sensors to some extent. As an excellent platform merging fibre optics and nanotechnology, MNF optics will continue to open up new opportunities in broad areas ranging from nanophotonics, nonlinear optics to quantum optics, which may be readily applied for MNF-based optical sensing, as better micro-/nanosensors are increasingly demanded from scientific research to daily life. Also, the unique properties of the MNF, originated from its small size, extraordinary geometric and material uniformity, will continue to offer new opportunities in MNF-based optical sensing, and hope that some points in this paper can be helpful or realized in future studies.
